# Supplementary material for: Structure of the p53/RNA polymerase II assembly
Source: Commun Biol. 2021 Mar 25;4:397. doi: 10.1038/s42003-021-01934-4 (PMC7994806; doi:10.1038/s42003-021-01934-4)
Supplement: Supplementary file 2 — Supplementary Information [file 42003_2021_1934_MOESM2_ESM.pdf]

Supplementary Figure S1: Strategy for 3D reconstruction of p53/Pol II

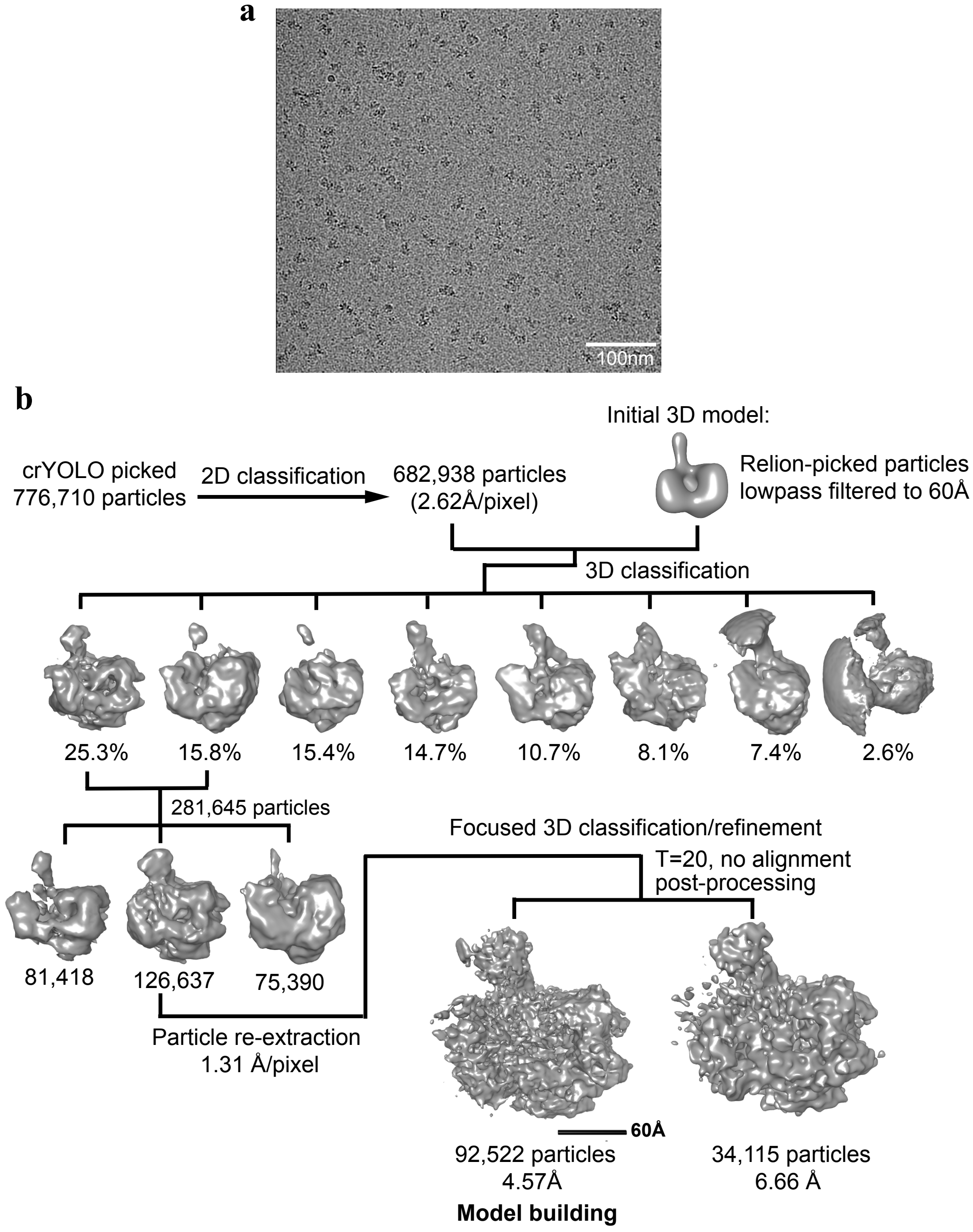

(a) A representative raw cryo-EM micrograph shows particles used for 3D reconstruction. The scale bar represents 100 nm. (b) Cryo-EM data processing strategy for 3D reconstruction of p53/Pol II using RELION-3.0[1] (see Materials and Methods). A RELION-reconstructed initial model of p53/Pol II (see Supplementary Methods) was applied for the 3D reconstruction of p53/Pol II using a total of 682,938 particles obtained from crYOLO[2]. Based on the initial unsupervised 3D classification, the first two 3D Classes resembling our previous 3D structure of p53/Pol II[3] were pooled and subjected to additional 3D classification. Particles from the dominant 3D class were used for focused 3D classification, 3D refinement and post-processing to generate the final reconstruction of p53/Pol II at a global resolution of 4.6 Å.

Supplementary Figure S2: 3D reconstruction of p53/Pol II

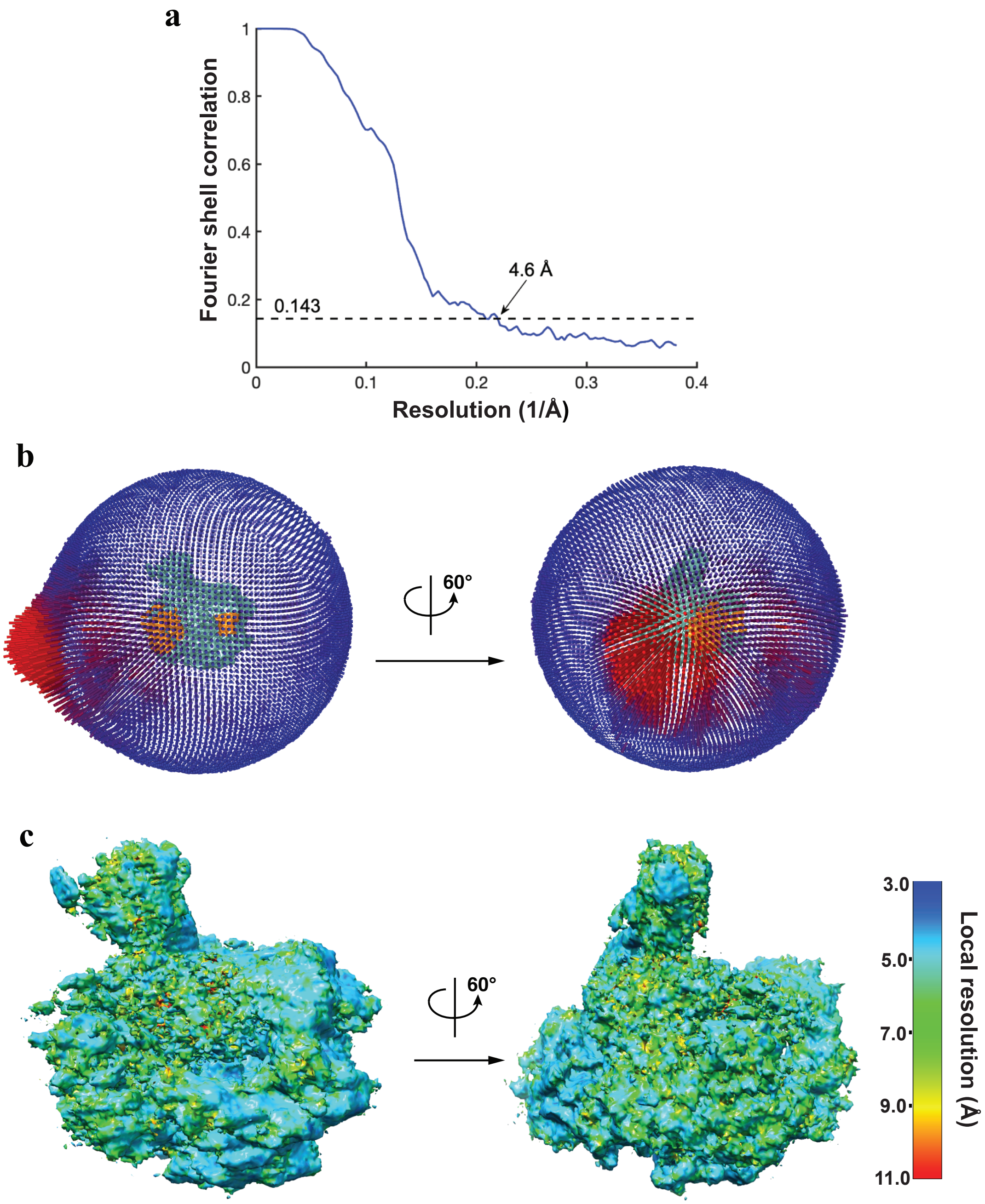

(a) A Fourier shell correlation (FSC) curve was generated in RELION-3.0[1] and the estimated global resolution was determined using the 0.143 criteria following the gold-standard procedure[4]. (b) Two views of the 3D plot showing Euler angle distribution of particles used in the final 3D reconstruction of p53/Pol II. Particle numbers are proportional to cylinder length, and are also indicated via cylinder color (purple which represents fewer particles, red which represents more particles). The entire 3D plot of Euler angle distribution is shown in the Supplementary Movie S2. (c) Local resolution of our cryo-EM density map was calculated using the Resmap software package[5]

Supplementary Figure S3: Structural conservation of the p53 core domain

a

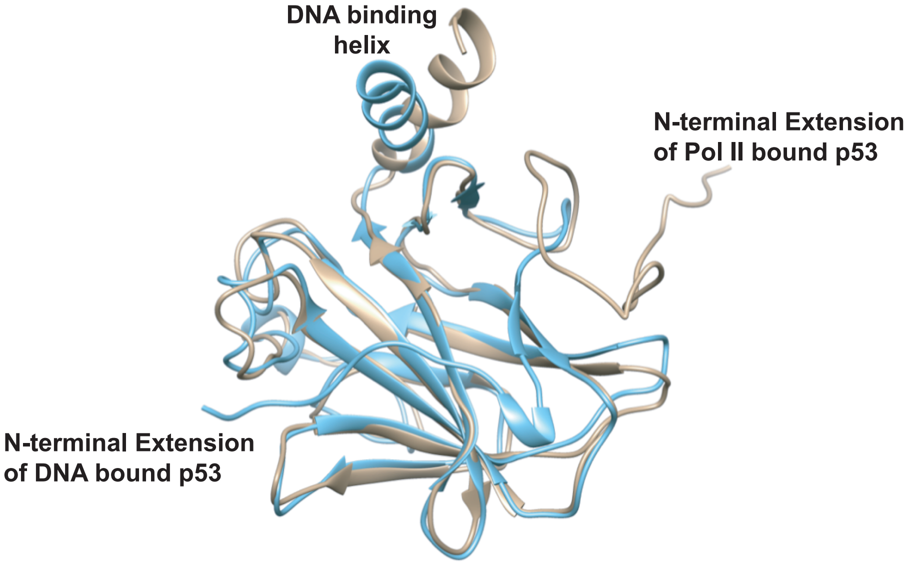

b

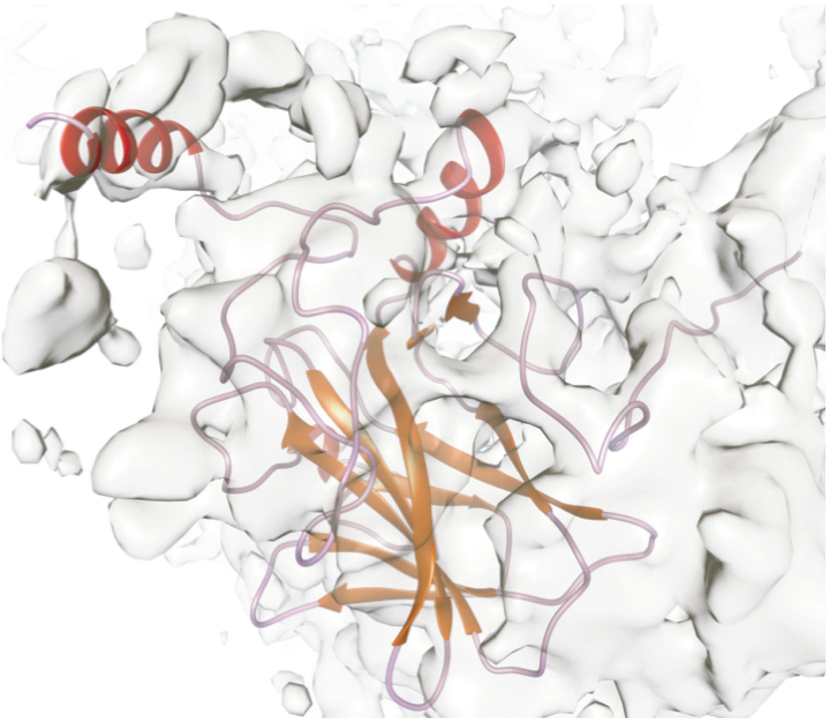

(a) Structural alignment of the p53 core domain bound to Pol II (gold, PDB:6XRE) and DNA (cyan, PDB:3TS8). (b) Model of p53 core (gold) and oligomerization (red) domains overlaid with the EM density from the p53/Pol II reconstruction.

Supplementary Figure S4: Model and structural conservation of the p53 TAD1/2 domain

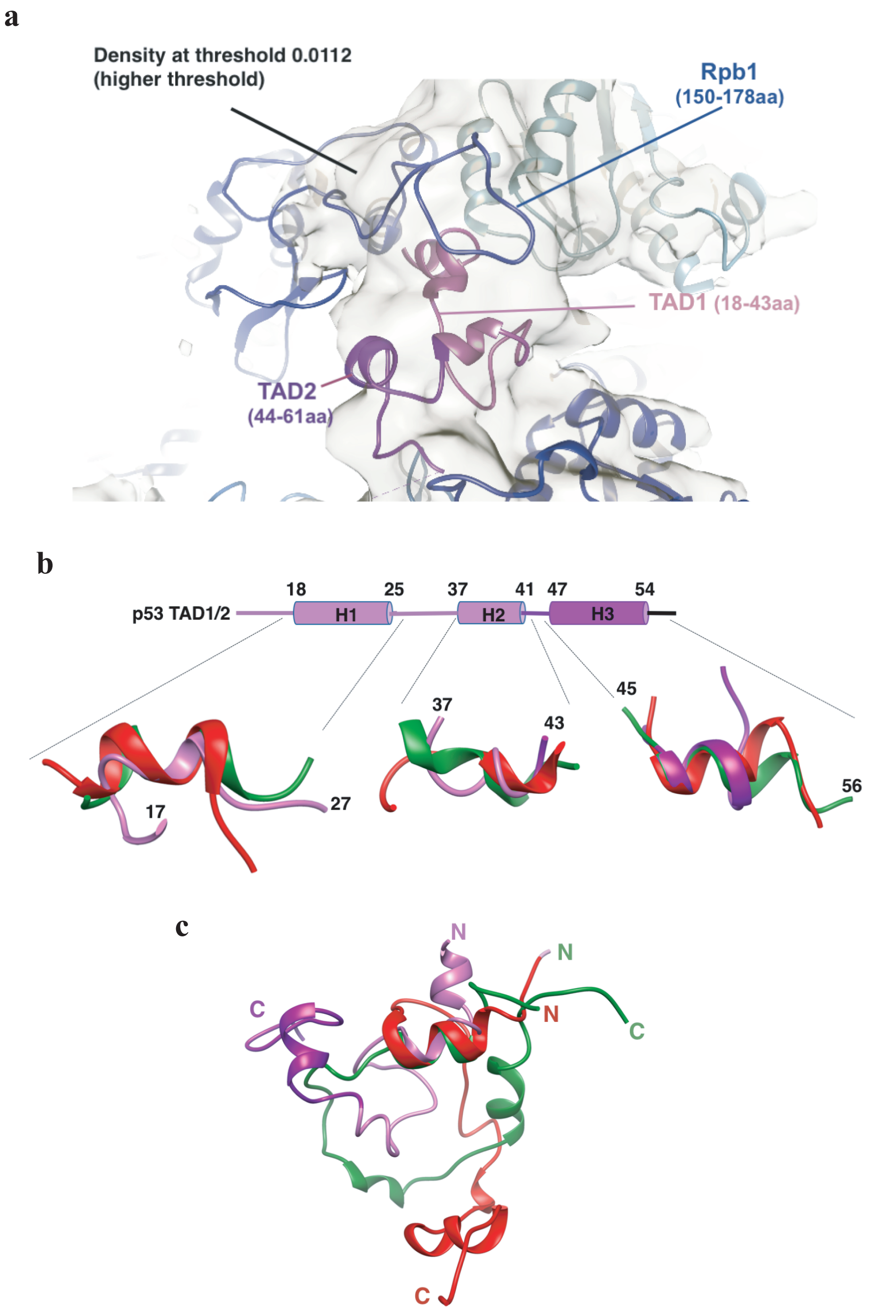

(a) Model of the p53 TAD1/2 domain (purple/magenta) overlaid with the EM density (gray) of the p53/Pol II reconstruction. The EM density threshold is at 0.0112. (b) Structural alignment of the individual helices of p53 TAD1/2 domain when bound to Pol II (purple/magenta, PDB:6XRE), the CBP nuclear coactivator binding domain (NCBD) (green, PDB:2L14) and the CBP TAZ1 domain (red, PDB:5HOU). (c) Structural alignment focused on Helix 1 of TAD1/2 showing the overall folding of the 3 TAD1/2 helices when bound to Pol II (purple/magenta) along with the CBP NCBD (green) and CBP TAZ1 (red) domains. In panels B and C, protein domains of Pol II and CBP that interact with the p53 TAD1/2 domain have been omitted for clarity.

Supplementary Figure S5: Multibody refinement of the Pol II clamp region

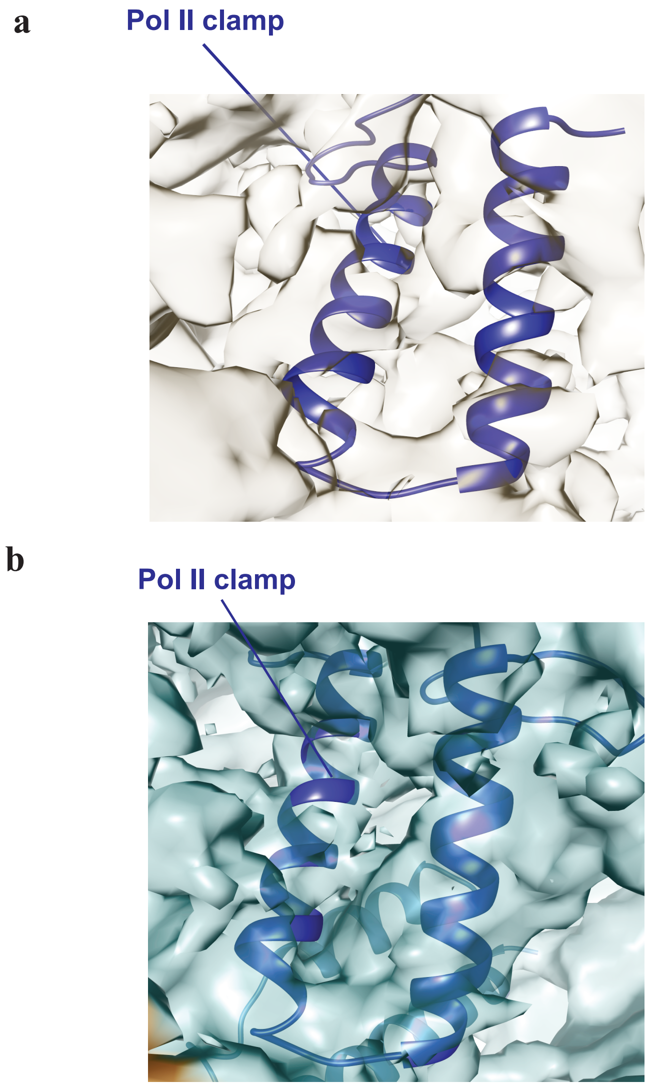

Model of the Pol II clamp (blue) overlaid with the EM density (gray) of the p53/Pol II reconstruction before (a) and after (b) multibody refinement in RELION.

Supplementary Figure S6: Model for recruitment of a p53/Pol II co-complex to DNA

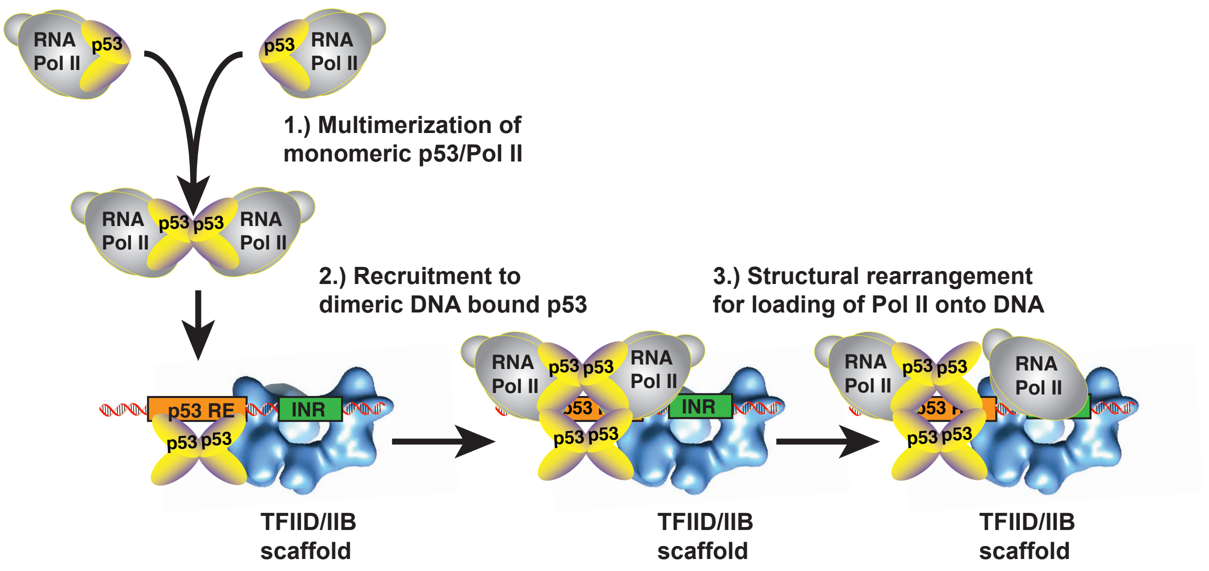

In our hypothetical model, a monomeric p53/Pol II co-complex multimerizes via dimerization of p53 subunits. The multimeric p53/Pol II co-complex can then assemble onto dimeric p53 bound to a p53 Response Element (p53 RE) that is interacting with a TFIID/IIB assembly on core promoter DNA. An ensuing conformational change in the p53/Pol II interface allows Pol II binding to the Initiator element (INR) DNA for subsequent PIC assembly.

Supplementary Methods

Generation of initial 3D reference model for 3D reconstruction of p53/Pol II

The same set of micrographs was used for data processing and 3D reconstruction of p53/Pol II exclusively via RELION-3.0 as described below[1]. Approximately 5,500 movies were acquired and further binned by 2-fold to 1.31 Å/pixel for faster data storage, and drift-corrected by MotionCor2[6] (Supplementary Figure S1a). Contrast transfer function parameters were calculated by Gctf[7]. Poor-quality and low-resolution micrographs were manually removed. A total of 2,257 particles were picked manually from five micrographs that represented the entire defocus spectra. These particles were then used to generate reference-free 2D class averages. These initial representative 2D classes were used as references to autopick particles from all micrographs, resulting in 1,094,141 particles. Eight sequential 2D classifications were performed to remove junk particles. The remaining 782,323 particles were applied for the initial 3D classification using our previous low-resolution p53/Pol II 3D structure (EMD-8538[3], with a 60 Å lowpass filter) as an initial model. Eight 3D classes were generated, and three classes with similar structural features were combined for another 3D classification with two classes. A total of 105,390 particles obtained from the dominant 3D class were further processed for 3D refinement, resulting in a global 7.9 Å resolution 3D reconstruction of p53/Pol II following the gold-standard resolution estimation (i.e. FSC curve at 0.143 value)[4].

Supplementary references

1 Zivanov, J. *et al.* New tools for automated high-resolution cryo-EM structure determination in RELION-3. *Elife* **7**, doi:10.7554/eLife.42166 (2018).

2 Wagner, T. *et al.* SPHIRE-crYOLO is a fast and accurate fully automated particle picker for cryo-EM. *Commun Biol* **2**, 218, doi:10.1038/s42003-019-0437-z (2019).

3 Singh, S. K. *et al.* Structural visualization of the p53/RNA polymerase II assembly. *Genes Dev* **30**, 2527-2537, doi:10.1101/gad.285692.116 (2016).

4 Scheres, S. H. & Chen, S. Prevention of overfitting in cryo-EM structure determination. *Nat Methods* **9**, 853-854, doi:10.1038/nmeth.2115 (2012).

5 Kucukelbir, A., Sigworth, F. J. & Tagare, H. D. Quantifying the local resolution of cryo-em density maps. *Nat Methods* **11**, 63-65, doi:doi.org/10.1038/nmeth.2727 (2014).

6 Zheng, S. Q. *et al.* MotionCor2: anisotropic correction of beam-induced motion for improved cryo-electron microscopy. *Nature Methods* **14**, 331-332, doi:10.1038/nmeth.4193 (2017).

7 Zhang, K. Gctf: Real-time CTF determination and correction. *J Struct Biol* **193**, 1-12, doi:10.1016/j.jsb.2015.11.003 (2016).
