# Supplementary material for: Structure of the p53/RNA polymerase II assembly
Source: Commun Biol. 2021 Mar 25;4:397. doi: 10.1038/s42003-021-01934-4 (PMC7994806; doi:10.1038/s42003-021-01934-4)
Supplement: Supplementary file 3 — Description of Additional Supplementary Files [file 42003_2021_1934_MOESM3_ESM.pdf]

Description of Additional Supplementary Files

File Name: Supplementary Video 1

Description: The structural alignment of p53-Pol II cryo-EM density map and its molecular structure

File Name: Supplementary Video 2

Description: Local resolution and Euler angle distributions of p53-Pol II
