# Supplementary material for: Structure of the p53/RNA polymerase II assembly
Source: Commun Biol. 2021 Mar 25;4:397. doi: 10.1038/s42003-021-01934-4 (PMC7994806; doi:10.1038/s42003-021-01934-4)
Supplement: Supplementary file 6 — Reporting Summary [file 42003_2021_1934_MOESM6_ESM.pdf]

nature research

Corresponding author(s): Wei-Li Liu & Robert A. Coleman

Last updated by author(s): Feb 6, 2021

Reporting Summary

Nature Research wishes to improve the reproducibility of the work that we publish. This form provides structure for consistency and transparency in reporting. For further information on Nature Research policies, see our Editorial Policies and the Editorial Policy Checklist.

Statistics

For all statistical analyses, confirm that the following items are present in the figure legend, table legend, main text, or Methods section.

n/a	Confirmed	
☐	☒	The exact sample size (*n*) for each experimental group/condition, given as a discrete number and unit of measurement
☐	☒	A statement on whether measurements were taken from distinct samples or whether the same sample was measured repeatedly
☒	☐	The statistical test(s) used AND whether they are one- or two-sided<br>*Only common tests should be described solely by name; describe more complex techniques in the Methods section.*
☒	☐	A description of all covariates tested
☒	☐	A description of any assumptions or corrections, such as tests of normality and adjustment for multiple comparisons
☒	☐	A full description of the statistical parameters including central tendency (e.g. means) or other basic estimates (e.g. regression coefficient) AND variation (e.g. standard deviation) or associated estimates of uncertainty (e.g. confidence intervals)
☒	☐	For null hypothesis testing, the test statistic (e.g. *F*, *t*, *r*) with confidence intervals, effect sizes, degrees of freedom and *P* value noted<br>*Give P values as exact values whenever suitable.*
☒	☐	For Bayesian analysis, information on the choice of priors and Markov chain Monte Carlo settings
☒	☐	For hierarchical and complex designs, identification of the appropriate level for tests and full reporting of outcomes
☒	☐	Estimates of effect sizes (e.g. Cohen's *d*, Pearson's *r*), indicating how they were calculated

Our web collection on statistics for biologists contains articles on many of the points above.

Software and code

Policy information about availability of computer code

Data collection	SerialEM (3.7)
Data analysis	crYOLO 1.6.1, Relion-3.0, Gctf 1.06, MotionCor2-1, UCSF Chimera 1.14, Coot 0.8.9.2, Phenix 1.17.1, Rosetta 3.9, Resmap 1.1.4, Amber 16, Pymol 2.3.4

For manuscripts utilizing custom algorithms or software that are central to the research but not yet described in published literature, software must be made available to editors and reviewers. We strongly encourage code deposition in a community repository (e.g. GitHub). See the Nature Research guidelines for submitting code & software for further information.

Data

Policy information about availability of data

All manuscripts must include a data availability statement. This statement should provide the following information, where applicable:

- Accession codes, unique identifiers, or web links for publicly available datasets
- A list of figures that have associated raw data
- A description of any restrictions on data availability

The molecular structure and cryo-EM density map will be available from the RCSB Protein Data Bank (6XRE) and Electron Microscopy Database (EMD-22294). We have placed the data on hold to be released upon publication.

Field-specific reporting

Please select the one below that is the best fit for your research. If you are not sure, read the appropriate sections before making your selection.

☒ Life sciences ☐ Behavioural & social sciences ☐ Ecological, evolutionary & environmental sciences

For a reference copy of the document with all sections, see nature.com/documents/nr-reporting-summary-flat.pdf

Life sciences study design

All studies must disclose on these points even when the disclosure is negative.

Sample size	We collected 5500 micrographs. cryo-EM of p53/Pol II complexes results utilized approximately 776,710 particles.
Data exclusions	The micrographs with bad ice are excluded as common practice in cryo-EM field.
Replication	Our early publication with different particle set results in ~11A resolution density map, which is similar as current results. In addition, we have conducted two different approaches of particle picking that results in similar density map but with different resolution.
Randomization	Relion adapted unbiased particle classification (maximum likelihood) which include randomizations. In addition, the particles were randomized during the analysis.
Blinding	Blinding was not applicable.

Reporting for specific materials, systems and methods

We require information from authors about some types of materials, experimental systems and methods used in many studies. Here, indicate whether each material, system or method listed is relevant to your study. If you are not sure if a list item applies to your research, read the appropriate section before selecting a response.

Materials & experimental systems

n/a	Involved in the study	
☐	☒	Antibodies
☐	☒	Eukaryotic cell lines
☒	☐	Palaeontology and archaeology
☒	☐	Animals and other organisms
☒	☐	Human research participants
☒	☐	Clinical data
☒	☐	Dual use research of concern

Methods

n/a	Involved in the study	
☒	☐	ChIP-seq
☒	☐	Flow cytometry
☒	☐	MRI-based neuroimaging

Antibodies

Antibodies used	8WG16 anti-Pol II C-terminal domain (CTD) monoclonal antibody (can be purchased from Sigma-Aldrich cat 05-952-I)
Validation	From Sigma-Aldrich's website: Anti-RNA Polymerase II, CTD, clone 8WG16, Cat. No. 05-952-I, is a mouse monoclonal antibody that detects DNA-directed RNA polymerase II subunit RPB1 and has been tested for use in Dot Blot, Chromatin Immunoprecipitation, Immunoprecipitation, Inhibition assay, and Western Blotting

Eukaryotic cell lines

Policy information about cell lines

Cell line source(s)	Standard HeLa cells from the Tjian Lab at UC-Berkeley obtained from ATCC
Authentication	cell line was not authenticated
Mycoplasma contamination	cell lines were not tested for Mycoplasma
Commonly misidentified lines (See ICLAC register)	N/A
